# Exploring human–artificial intelligence interactions in a negative pragmatic trial of computer-aided polyp detection

**DOI:** 10.1016/j.igie.2024.04.016

**Published:** 2024-05-04

**Authors:** Kate Watkins, Uri Ladabaum, Esther Olsen, Jonathan Hoogerbrug, Ajitha Mannalithara, Yingjie Weng, Blake Shaw, Roger Bohn, Sara Singer

**Affiliations:** 1 Department of Health Policy, Stanford University School of Medicine, Stanford, California, USA; 2 Division of Gastroenterology and Hepatology, Stanford University School of Medicine, Stanford, California, USA; 3 Clinical Excellence Research Center, Stanford University School of Medicine, Stanford, California, USA; 4 Quantitative Sciences Unit, Stanford University School of Medicine, Stanford, California, USA; 5 School of Global Policy and Strategy, University of California–San Diego, San Diego, California, USA

## Abstract

**Background and Aims:**

The progress of artificial intelligence (AI) in endoscopy is at a crossroads. The positive results of randomized controlled trials of computer-aided detection (CADe) have not been replicated in multiple pragmatic CADe trials, including ours. This gap between efficacy and effectiveness remains to be understood. We surveyed and interviewed our trial’s colonoscopists to gain insight into human-AI interactions.

**Methods:**

We used a sequential, mixed-methodology design. After the trial, we administered Survey 1, focusing on attitudes and beliefs before and after trying CADe. The trial’s null results were disclosed, and we then administered Survey 2 and conducted open-ended interviews, focusing on reactions to the null results. Responses were analyzed overall and by baseline adenoma detection rate (ADR) tertile. We identified key themes using thematic analysis and qualitative software.

**Results:**

Nearly all colonoscopists responded (22 and 21 of 24 [92% and 88%] for Surveys 1 and 2, respectively). Most (96%) regarded endoscopic ability as critical to their professional identity. Large majorities conveyed trust in and enthusiasm for AI before and after trying CADe (82%-87%) and desired to have CADe available (72%). Nearly two-thirds (62%) were surprised by the null results. There were few differences by ADR. No unifying explanation for the null results emerged from surveys or individual interviews. Colonoscopists expressed a range of expectations for AI in endoscopy.

**Conclusions:**

Lack of enthusiasm or mistrust of AI/CADe do not explain our pragmatic CADe trial’s null results. AI may need to target dimensions beyond optical recognition to realize its promise in endoscopy.

Artificial intelligence (AI) could revolutionize endoscopy.^1^, ^2^, ^3^ Computer-aided detection (CADe) emerged with great enthusiasm as the first viable application of AI in colonoscopy. Polyp detection is an ideal target for CADe because a colonoscopist’s adenoma detection rate (ADR) is inversely associated with patients’ risks of postcolonoscopy colorectal cancer (CRC) incidence and death,^4^, ^5^, ^6^ and CADe could overcome human errors in visual detection.

The progress of AI in colonoscopy is now at a crossroads. Multiple randomized controlled trials (RCTs) have shown substantial improvements in ADR, adenomas per colonoscopy, and sessile serrated lesion (SSL) detection rate with CADe.^7^, ^8^, ^9^ However, we now confront a gap between the efficacy demonstrated in the RCTs and the minimal or absent effectiveness observed in real-world data,^10^, ^11^, ^12^, ^13^, ^14^, ^15^, ^16^, ^17^ as summarized in 2 recent meta-analyses.^18^^,^^19^ In the RCTs, blinding was not possible, and colonoscopies were often done by experienced endoscopists who were also authors of the publications. It remains to be determined whether these features of the RCTs could explain, at least in part, their better results compared to most of the published real-world experience.

Our pragmatic implementation trial^20^ of a CADe platform with encouraging RCT results^21^, ^22^, ^23^ was one of the first real-world studies that failed to replicate the benefits seen in RCTs. The colonoscopists who participated in that trial are an ideal study population with which to explore the potential reasons for the current gap between the efficacy and effectiveness of CADe.

Upon learning of the null results of our trial, we partnered with social scientists with expertise in technology implementation and launched a mixed-methodology study to develop a deeper understanding of human-AI interactions, seek explanations for the null trial results, and identify directions for future research. We surveyed and interviewed the colonoscopists who participated in our pragmatic trial to explore attitudes and beliefs related to AI and CADe before and after their clinical experience with CADe as well as their reactions and thoughts regarding the disappointing results of our pragmatic trial. We achieved a very high participation rate, so our results provide a comprehensive assessment of the trial’s colonoscopist group as a whole.

## Methods

### Study design, setting, and participants

We used a sequential, mixed-methodology design^24^ (see Appendix 1 for full methodology, available online at www.igiejournal.org) to understand Stanford colonoscopists’ experience with CADe (GI Genius Intelligent Endoscopy Module, Medtronic, Minneapolis, Minn, USA) and to explore potential explanations for the lack of improvement in quality metrics in our pragmatic CADe trial.^20^ We did not survey colonoscopists before or during the trial given our explicit intent to avoid any undue influence on performance. Stanford University’s institutional review board approved this study.

We administered 2 surveys, the first following the pragmatic trial (Survey 1) and the second following disclosure of our pragmatic trial results at a faculty meeting (Survey 2). We then conducted qualitative interviews that built on the survey results to gain a deeper understanding of colonoscopists’ reactions and the pragmatic trial results.

For the surveys, we invited all 24 colonoscopists who participated in the CADe pragmatic trial.^20^ Participation was voluntary, and respondents received no remuneration. Based on our quality assurance program’s routine comprehensive audit,^25^, ^26^, ^27^, ^28^ we divided the 24 colonoscopists into tertiles based on their baseline ADR performance in the 12 months preceding the CADe trial.^20^ Colonoscopists were assigned blinded identifiers (IDs), and the key was not disclosed to the other authors and was not consulted again, as described previously.^26^ For the interviews, we continued inviting colonoscopists until we reached data saturation, where ideas expressed repeated those from prior interviews.^29^

### Surveys 1 and 2

We developed our surveys based on the “Survey on the Future of Technology-Assisted Work,” which the social scientists developed previously to study beliefs and attitudes toward technology in the intensive care setting (available upon request).^30^ Survey 1 (Appendix 1) included 33 questions probing attitudes and beliefs about CADe. Survey 2 (Appendix 1) included 8 questions about colonoscopists’ reactions to and explanations for the CADe pragmatic trial results.

### Survey data analysis

For most items, responses were recorded on a 5-point Likert scale for the degree of respondent agreement to each item, ranging from strongly disagree (1) to strongly agree (5). For each item, we calculated the percentage of responses for each Likert scale option. We linked deidentified, coded survey data with the corresponding baseline ADR tertile for each respondent and compared results by tertile using the Kruskal-Wallis test. Complete case analysis was performed. A *P* value of <.05 was considered statistically significant for the descriptive analysis. The analysis was performed using SAS software version 9.4 (SAS Institute Inc, Cary, NC, USA).

### Interview guide, data collection, and analysis

Building on survey findings, we developed a semistructured interview guide, consisting of 8 questions (Appendix 1), to probe endoscopists’ reactions to the use of CADe in endoscopy and to explore potential explanations for the trial results. We performed individual interviews on Zoom (Zoom Video Communications, San Jose, Calif, USA). With participant consent, we recorded, transcribed, and deidentified the interviews. Data were analyzed according to the principles of thematic analysis, combining deductive and inductive approaches.^31^

## Results

### Survey 1: Professional identity, beliefs, and attitudes toward AI and trust in CADe

Of the 24 colonoscopists in the CADe trial, 22 (92%) responded to Survey 1, and 21 of 22 (96%) agreed or strongly agreed that endoscopic ability is critical to their professional identity.

**Figure 1:**
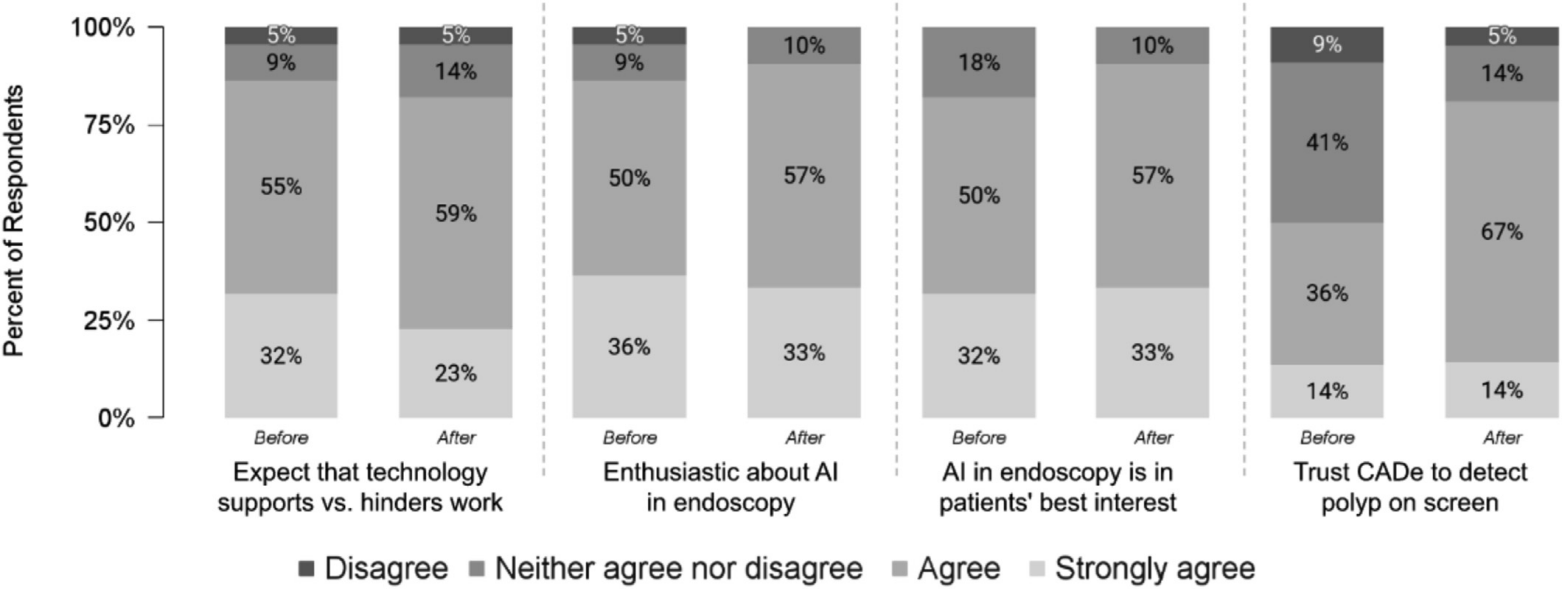
Survey 1: beliefs, and attitudes toward AI and trust in CADe before and after trying CADe. Scale options included “strongly disagree,” but no respondent selected that option. *AI*, Artificial intelligence; *CADe*, computer-aided detection.

Before trying CADe, most (82%-87%) agreed or strongly agreed that AI technology would support rather than hinder their work, were enthusiastic about the use of AI/CADe in endoscopy, and believed that applying AI in endoscopy is in the best interest of patients; 50% trusted CADe to detect polyps that are displayed on the screen (Fig. 1 and Table 1).

**Table 1:** Survey 1: Beliefs and attitudes before and after trying CADe, overall and by baseline ADR tertile

Belief/attitude	n (%)					Mean (SD)				*P* value
	Strongly agree (5)	Agree (4)	Neither agree nor disagree (3)	Disagree (2)	Strongly disagree (1)	Overall	Bottom ADR tertile	Middle ADR tertile	Top ADR tertile	
Before trying the CADe (GI Genius) module										
My experience with new technologies at work generally led me to expect that technology will support rather than hinder my work	7 (32)	12 (55)	2 (9)	1 (5)	–	4.1 (0.8)	4.4 (0.8)	4.1 (0.7)	3.9 (0.8)	.36
I was enthusiastic about the application of artificial intelligence (AI) in endoscopy	8 (36)	11 (50)	2 (9)	1 (5)	–	4.2 (0.8)	4.3 (0.8)	4.4 (0.5)	3.9 (1.0)	.49
I believed that applying artificial intelligence (AI) in endoscopy is in the best interest of patients	7 (32)	11 (50)	4 (18)	–	–	4.1 (0.7)	4.3 (0.8)	4.1 (0.7)	4.0 (0.8)	.74
I trusted the CADe (GI Genius) module to detect polyps that are displayed on the screen	3 (14)	8 (36)	9 (41)	2 (9)	–	3.6 (0.9)	3.9 (0.9)	3.3 (1.1)	3.5 (0.5)	.54
I regarded my abilities in endoscopy as a critical part of my professional identity	15 (68)	6 (27)	1 (5)	–	–	4.6 (0.6)	4.4 (0.5)	4.6 (0.8)	4.9 (0.4)	.23
After trying the CADe (GI Genius) module										
I believe this technology can support rather than hinder my work	5 (23)	13 (59)	3 (14)	1 (5)	–	4.0 (0.8)	3.6 (1.0)	4.0 (0.6)	4.4 (0.5)	.15
I am enthusiastic about the application of artificial intelligence (AI) in endoscopy	7 (33)	12 (57)	2 (10)	–	–	4.2 (0.6)	4.2 (0.4)	4.1 (0.7)	4.4 (0.7)	.66
I believe that applying artificial intelligence (AI) in endoscopy is in the best interest of patients	7 (33)	12 (57)	2 (10)	–	–	4.2 (0.6)	4.0 (0.6)	4.4 (0.5)	4.3 (0.7)	.48
I trust the CADe (GI Genius) module to detect polyps that are displayed on the screen	3 (14)	14 (67)	3 (14)	1 (5)	–	3.9 (0.7)	4.0 (0.6)	4.0 (0.6)	3.8 (0.9)	.85
I received adequate training on the use of the CADe (GI Genius) module	6 (30)	10 (50)	2 (10)	2 (10)	–	4.0 (0.9)	4.0 (1.1)	4.0 (0.6)	4.0 (1.1)	.93
The CADe (GI Genius) module was easy to use	12 (57)	9 (43)	–	–	–	4.6 (0.5)	4.5 (0.5)	4.4 (0.5)	4.8 (0.5)	.43
The CADe (GI Genius) “green boxes” when there was not really a polyp were bothersome	3 (14)	6 (29)	5 (24)	7 (33)	–	3.2 (1.1)	3.0 (1.1)	3.3 (1.0)	3.4 (1.3)	.83
The CADe (GI Genius) sound that went along with the “green box” was bothersome	3 (15)	5 (25)	9 (45)	3 (15)	–	3.4 (0.9)	3.3 (1.2)	3.7 (0.8)	3.3 (0.9)	.67
The CADe (GI Genius) module improved my overall performance as a colonoscopist	1 (5)	8 (38)	11 (52)	1 (5)	–	3.4 (0.7)	3.3 (0.5)	3.4 (0.5)	3.5 (0.9)	.90
The CADe (GI Genius) module FOUND a clinically meaningful number of polyps that I missed	1 (5)	1 (5)	11 (52)	8 (38)	–	2.8 (0.8)	2.8 (0.8)	2.4 (0.5)	3.0 (0.9)	.36
The CADe (GI Genius) module MISSED a clinically meaningful number of polyps that I found	–	2 (10)	11 (52)	8 (38%	–	2.7 (0.6)	3.0 (0.9)	2.4 (0.5)	2.8 (0.5)	.33
The CADe (GI Genius) module improved my lesion detection rates during colonoscopy	1 (5)	7 (33)	10 (48)	3 (14)	–	3.3 (0.8)	3.0 (0.6)	3.4 (0.5)	3.4 (1.1)	.54
The CADe (GI Genius) module made me focus on exposing all the colonic mucosa better	2 (10)	8 (38)	8 (38)	3 (14)	–	3.4 (0.9)	3.3 (0.8)	3.6 (1.0)	3.4 (0.9)	.86
I would like to have the CADe (GI Genius) module that we trialed available for all my colonoscopies	6 (29)	9 (43)	6 (29)	–	–	4.0 (0.8)	4.0 (0.9)	3.7 (0.5)	4.3 (0.9)	.41
I would like to have artificial intelligence (AI) applications available for all my colonoscopies	8 (38)	10 (48)	3 (14)	–	–	4.2 (0.7)	4.3 (0.5)	4.0 (0.6)	4.4 (0.9)	.45
I am concerned that monitoring colonoscopy quality metrics may be used against me	2 (10)	4 (19)	11 (52)	4 (19)	–	2.2 (0.9)	2.3 (1.0)	2.1 (0.9)	2.1 (0.8)	.92
I worry that technology will replace me in doing important aspects of my work	–	2 (10)	1 (5)	12 (57)	6 (29)	2.0 (0.9)	2.2 (1.0)	2.0 (1.0)	1.8 (0.7)	.72

There were 22 respondents, but some items had missing responses, and therefore not all items have 22 responses. The percentages shown are relative to the number of respondents for each individual item.

*ADR*, Adenoma detection rate; *AI*, artificial intelligence; *CADe*, computer-aided detection; *SD*, standard deviation.

**Figure 2:**
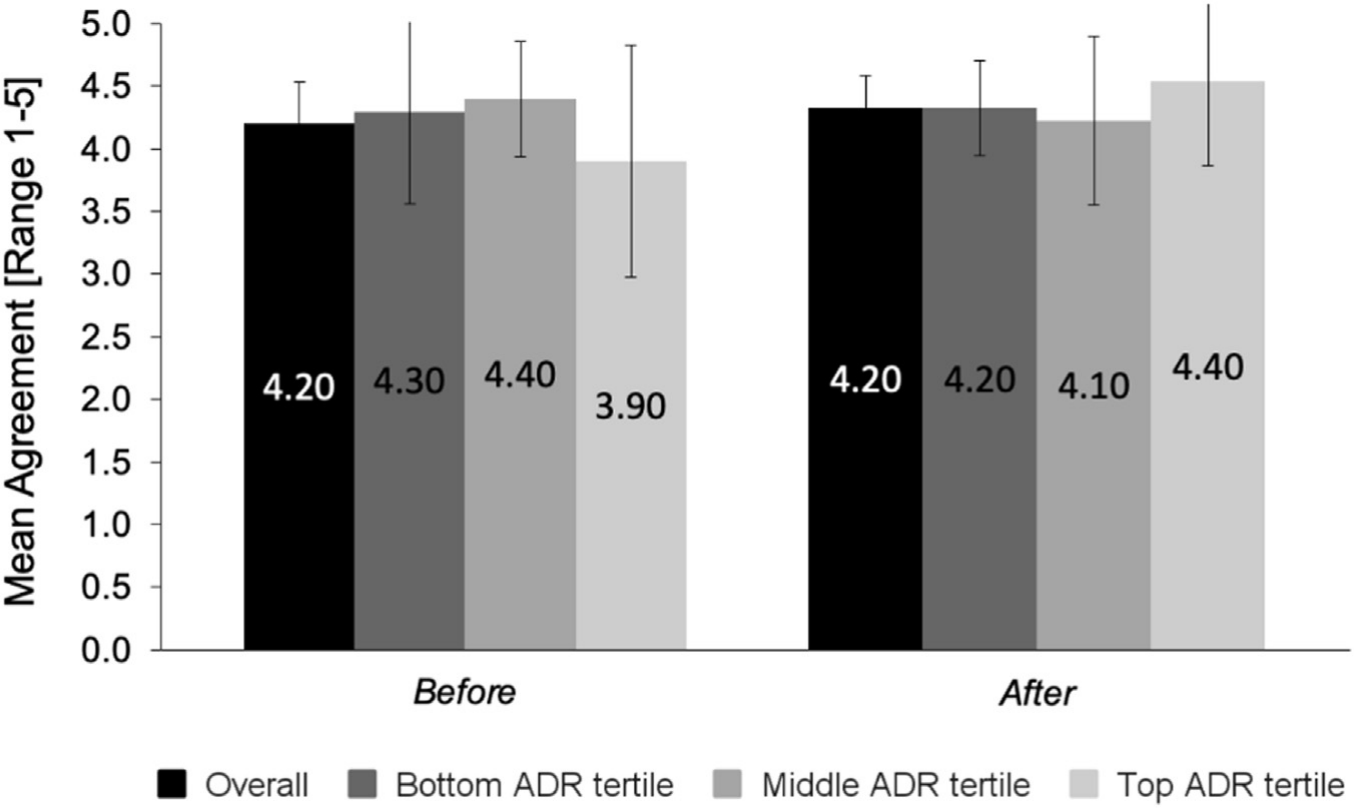
Survey 1: respondent enthusiasm about AI in endoscopy by baseline ADR performance tertile before and after trying CADe. Respondents were asked the extent to which they agreed or disagreed before and after trying CADe with the statement, “I [was/am] enthusiastic about the application of artificial intelligence (AI) in endoscopy.” Mean agreement was calculated as the average response on a scale of 1 to 5, where 1 = strongly disagree and 5 = strongly agree. The levels of enthusiasm for CADe did not vary significantly across ADR tertiles for any item before (*P* = .49) or after (*P* = .66) trying CADe. *ADR*, Adenoma detection rate; *AI*, artificial intelligence; *CADe*, computer-aided detection.

Responses were similar after trying CADe (Fig. 1 and Table 1). Trust in GI Genius to detect polyps that are displayed on the screen improved after trying CADe, but the difference did not reach statistical significance (*P* = .09). The enthusiasm for CADe did not vary significantly across ADR tertiles for any item before (*P* = .49) or after (*P* = .66) trying CADe (Fig. 2).

### Survey 1: Beliefs and attitudes regarding the CADe pragmatic trial before learning its results

**Figure 3:**
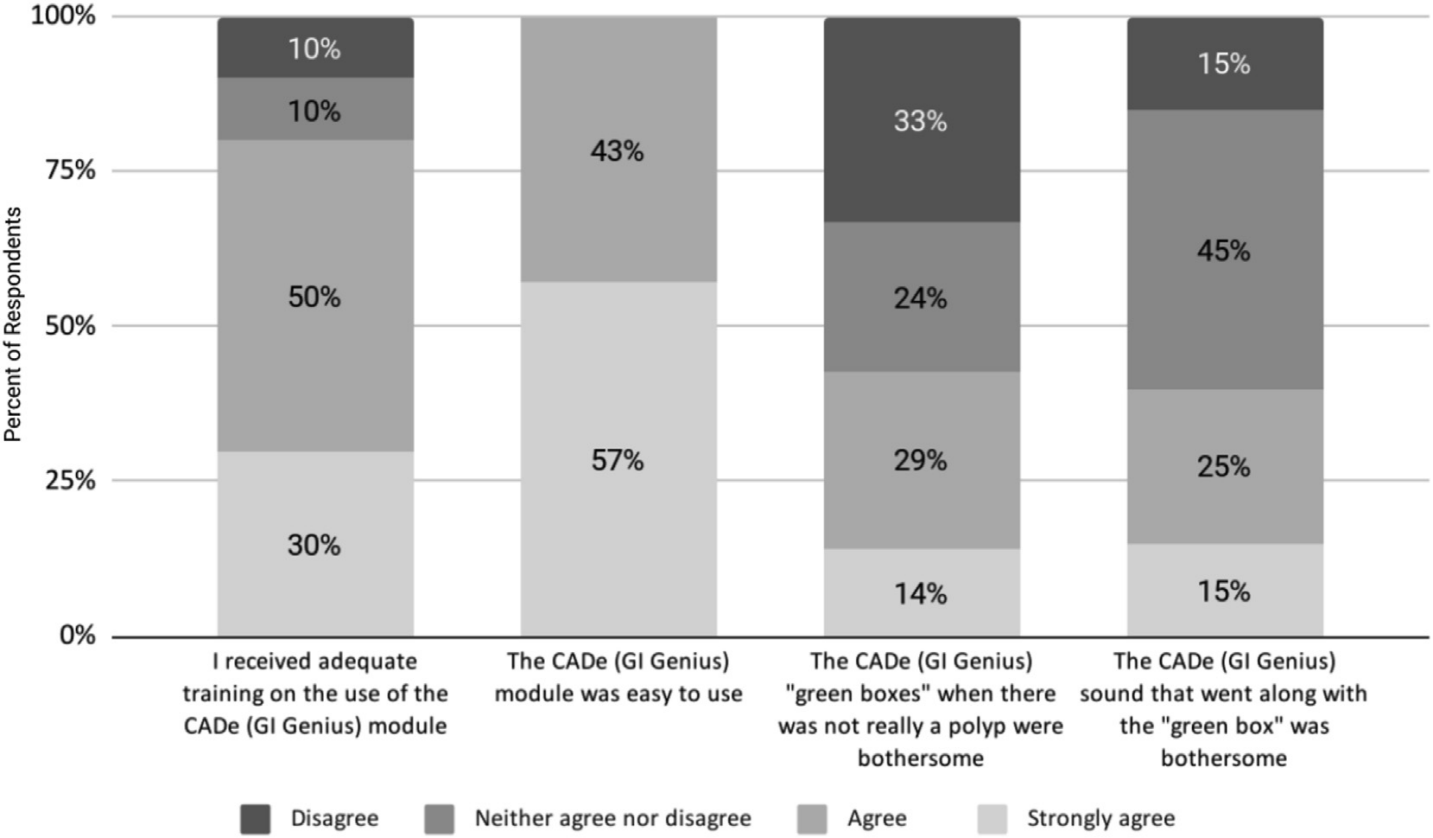
Survey 1: experience with the CADe module. Scale options included “strongly disagree,” but no respondent selected that option. *CADe*, Computer-aided detection.

Of the 22 survey respondents, 80% agreed or strongly agreed that they had received adequate training on CADe (Fig. 3 and Table 1). Despite perceived ease of use, slightly less than half (40%-43%) found the visual (“green box”) and auditory components of the automated polyp detection system bothersome (Fig. 3), leading 12 (57%) to disable the sound.

Before reviewing the group’s null pragmatic trial results, slightly less than half (43%) of respondents agreed or strongly agreed that CADe improved their overall performance, and a majority (72%) would like the same CADe module available for all colonoscopies (Table 1). Nearly half (48%) of respondents believed that CADe enhanced their focus on better exposing all the colonic mucosa, and more than a third (38%) believed it improved their lesion detection rates. Two (10%) believed CADe found a significantly meaningful number of polyps they had missed; 10% believed CADe missed a significantly meaningful number of polyps they had found (Table 1).

### Survey 1: Experience with the CADe module

When asked to expand on their experience with CADe, 75% of respondents provided additional comments. Notably, perceived strengths of the module included its ability to detect flat polyps and track polyps (Supplementary Table 1, available online at www.igiejournal.org) and to provide comfort by providing a “second pair of eyes” (44%). The most frequent concern (61%) was the frequent false positive for nonpolyp matter (eg, bubbles, Endocuff; Endocuff Vision, Olympus America, Center Valley, Pa, USA), with some (17%) reporting that they disliked the variability in CADe’s ability to detect polyps based on presentation (eg, more subtle polyps or flat polyps).

### Survey 1: Potential roles for AI in endoscopy

After trying CADe, most respondents (86%) wanted AI applications to be more available. When ranking potential roles for AI in endoscopy, moderate consensus emerged (Table 2). The most valuable role for AI (mean, 2.0; standard deviation [SD], 1.2) on a 1-to-6 scale (where 1 is most valuable) was assistance with ensuring adequate polyp detection during colonoscopy. The next most valuable were adequate polyp characterization during colonoscopy to guide real-time decisions about polyp resection (mean, 3.3; SD, 1.4) and ensuring adequate mucosal exposure (mean, 3.3; SD, 1.6). The top 3 most valuable applications of AI did not vary significantly by ADR tertile (Table 2). Participants’ suggestions for other applications are in Supplementary Table 2 (available online at www.igiejournal.org).

**Table 2:** Survey 1: Beliefs about potential roles for AI in endoscopy, overall and by baseline ADR tertile

Potential roles for AI	n (%)						Mean (SD)				*P* value
	Most valuable (1)	(2)	(3)	(4)	(5)	Least valuable (6)	Overall	Bottom ADR tertile	Middle ADR tertile	Top ADR tertile	
Assisting clinicians by predicting a patient’s clinical course or outcome (eg, cancer risk stratification or predicting risk of endoscopic adverse events)	2 (12)	1 (6)	2 (12)	3 (18)	2 (12)	7 (41)	4.4 (1.8)	3.8 (2.1)	4.0 (2.0)	5.0 (1.5)	.44
Assisting clinicians with ensuring adequate mucosal exposure during colonoscopy, through real-time feedback	2 (11)	5 (28)	5 (17)	3 (17)	3 (17)	2 (11)	3.3 (1.6)	3.5 (1.9)	3.7 (2.0)	3.0 (1.3)	.79
Assisting clinicians with ensuring adequate polyp detection during colonoscopy, through real-time feedback	9 (47)	6 (32)	1 (5)	2 (11)	1 (5)	–	2.0 (1.2)	2.0 (1.1)	2.0 (1.7)	1.9 (1.1)	.86
Assisting clinicians with ensuring adequate polyp characterization (eg, adenoma) during colonoscopy, through real-time feedback to guide real-time decisions about polyp resection	3 (17)	1 (6)	5 (28)	6 (33)	2 (11)	1 (6)	3.3 (1.4)	3.6 (1.8)	4.2 (0.8)	2.4 (1.1)	.047
Assisting clinicians with ensuring adequate polyp size determination during colonoscopy, through real-time feedback	–	4 (21)	2 (11)	4 (21)	7 (37)	2 (11)	4.1 (1.4)	3.6 (1.8)	4.0 (1.7)	4.4 (0.7)	.76
Assisting clinicians by automatic documentation of clinical activities (eg, endoscopy report generation)	4 (19)	3 (14)	5 (24)	2 (10)	2 (10)	5 (24)	3.5 (1.9)	3.8 (1.5)	2.9 (1.8)	3.8 (2.3)	.58

There were 22 respondents, but some items had missing responses, and therefore not all items have 22 responses. The percentages shown are relative to the number of respondents for each individual item. The survey instructions read as follows: “Listed below are 6 potential roles for Artificial Intelligence (AI) systems in endoscopy. Please RANK these potential roles from 1 (most valuable) to 6 (least valuable) based on the value that you would attach to each, assuming its benefit has been established through credible scientific research. Please use each ranking (ie, 1, 2, 3, 4, 5 and 6) ONLY ONCE.”

*ADR*, Adenoma detection rate; *AI*, artificial intelligence; *SD*, standard deviation.

### Survey 2: Understanding CADe trial results

**Figure 4:**
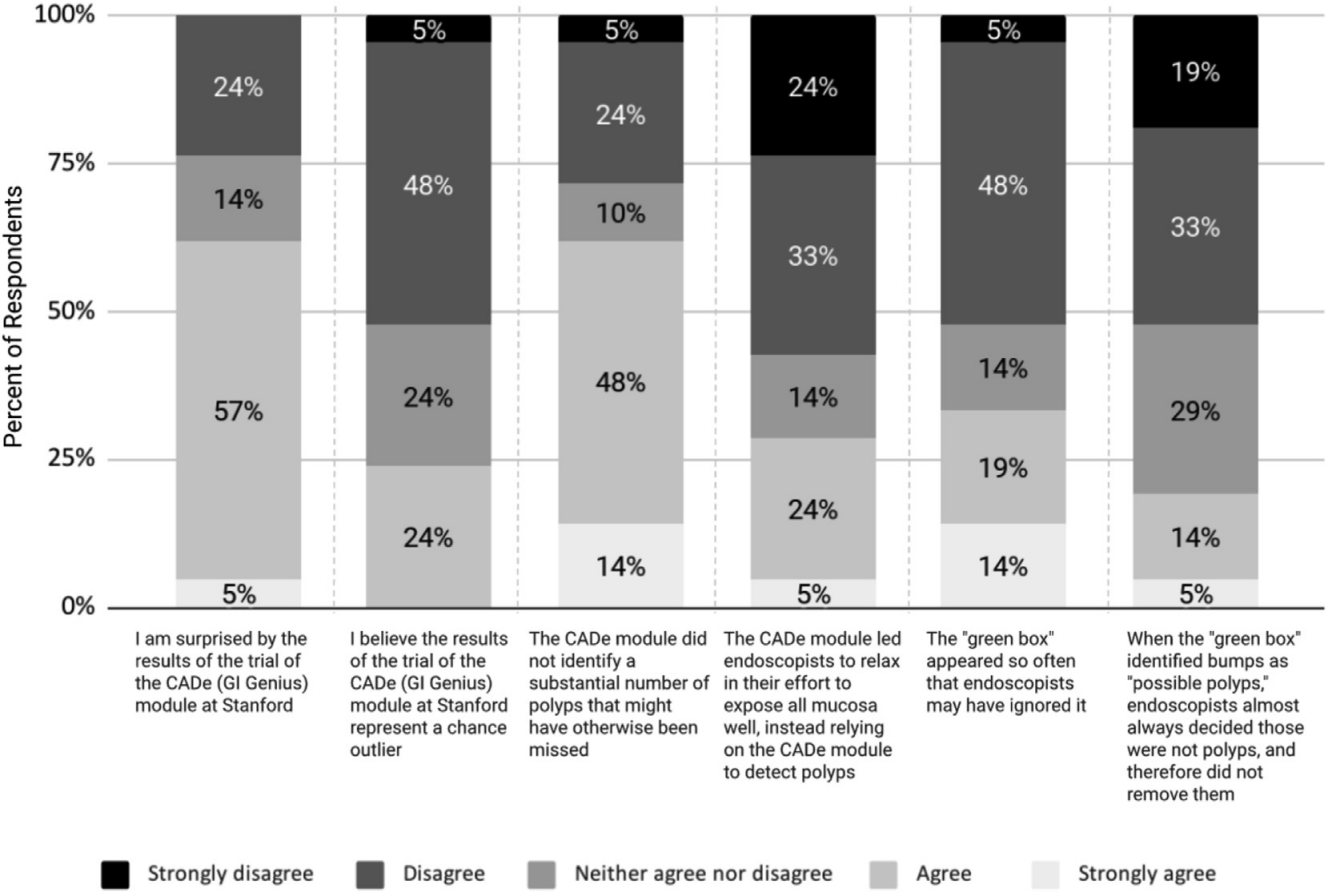
Survey 2: understanding the CADe trial results. *CADe*, Computer-aided detection.

A majority (62%) of the 21 colonoscopists (88% of the 24 in the CADe trial) who responded to Survey 2 were surprised by the pragmatic trial results, although almost a quarter (24%) believed that the results may represent a chance outlier (Table 3). There was little agreement among respondents about potential explanations for the trial results (Fig. 4). When provided with options and asked how strongly they agreed or disagreed, the majority (62%) agreed that CADe may not have found a substantial incremental number of polyps. Other explanations had less support (Fig. 4 and Table 3). Of note, significantly more higher- than lower-performing endoscopists believed that CADe may have led endoscopists to relax on mucosal exposure (*P* = .023). For other possible explanations, responses did not vary significantly across ADR tertiles (Table 3).

**Table 3:** Survey 2: Beliefs and attitudes after revealing the negative results of the CADe pragmatic trial, overall and by baseline ADR tertile

After the results of the open-label trial of CADe were revealed: To what extent do you agree or disagree with the following?	n (%)					Mean (SD)				*P* value
	Strongly agree (5)	Agree (4)	Neither agree nor disagree (3)	Disagree (2)	Strongly disagree (1)	Overall	Bottom ADR tertile	Middle ADR tertile	Top ADR tertile	
I am surprised by the results of the trial of the CADe (GI Genius) module at Stanford	1 (5)	12 (57)	3 (14)	5 (24)	–	3.4 (0.9)	3.3 (1.0)	3.5 (1.2)	3.5 (0.8)	.87
I believe the results of the trial of the CADe (GI Genius) module at Stanford represent a chance outlier (eg, maybe we colonoscoped people with fewer-than-average polyp numbers during the trial period)	–	5 (24)	5 (24)	10 (48)	1 (5)	2.7 (0.9)	2.7 (1.0)	2.8 (1.0)	2.5 (0.9)	.85
A possible explanation for the results of the trial of the CADe (GI Genius) module at Stanford is that the CADe module did not identify a substantial number of polyps that might have otherwise been missed	3 (14)	10 (48)	2 (10)	5 (24)	1 (5)	3.4 (1.2)	3.3 (1.3)	3.7 (1.4)	3.4 (1.1)	.76
A possible explanation for the results of the trial of the CADe (GI Genius) module at Stanford is that the CADe module led endoscopists to relax in their effort to expose all mucosa well, instead relying on the CADe module to detect polyps	1 (5)	5 (24)	3 (14)	7 (33)	5 (24)	2.5 (1.3)	2.4 (1.0)	1.5 (0.5)	3.4 (1.3)	.023
A possible explanation for the results of the trial of the CADe (GI Genius) module at Stanford is that the “green box" appeared so often that endoscopists may have ignored it	3 (14)	4 (19)	3 (14)	10 (48)	1 (5)	2.9 (1.2)	2.9 (1.5)	2.7 (0.8)	3.1 (1.4)	.81
A possible explanation for the results of the trial of the CADe (GI Genius) module at Stanford is that when the “green box” identified bumps as “possible polyps,” endoscopists almost always decided those were not polyps and therefore did not remove them	1 (5)	3 (14)	6 (29)	7 (33)	4 (19)	2.5 (1.1)	2.6 (1.3)	2.3 (1.0)	2.6 (1.2)	.85

There were 21 respondents to these items.

*ADR*, Adenoma detection rate; *CADe*, computer-aided detection; *SD*, standard deviation.

When invited to expand on the group’s null results through open-ended responses, 71% responded. Of these, 12% referenced Stanford’s “well-educated” patient population, 47% noted Stanford’s high average ADR compared to national standards, 5% highlighted the impact of the “annoying” sound, and 5% reported CADe’s inability to rectify procedural errors (eg, inadequate mucosal exposure) (Supplementary Table 3, available online at www.igiejournal.org).

### Interviews: Experience with CADe

We interviewed 11 colonoscopists who participated in the CADe trial, with similar representation of each baseline ADR tertile, after disclosure of the trial results. When no new themes emerged (data saturation), no further interviews were pursued. Each interview lasted approximately 20 minutes. The responses echoed and expanded on answers provided in the surveys.

As reported in Survey 1, respondents did not like the frequency of false positives, the perceived prolonged procedure time, and the potentially distracting nature of the “green box,” and they liked the comfort of having CADe as a “second set of eyes”; CADe’s ability to stabilize the colonoscope; and, for some respondents, the (friendly) competition to successfully identify polyps before the CADe (Supplementary Table 4, available online at www.igiejournal.org). Although colonoscopists generally felt neutral about CADe, several respondents noted disappointment that the CADe modules were removed at end of the pragmatic trial period.

### Interviews: Understanding the CADe trial results

Respondents volunteered several potential explanations for the pragmatic trial’s disappointing results (Supplementary Table 5, available online at www.igiejournal.org). The main themes reported in Survey 2 also emerged through interviews (specifically, Stanford’s patient population and high average ADR, the inability to correct procedural errors, and the user’s incorrect dismissal of the “green box” as a false positive). Additionally, one respondent postulated that Stanford colonoscopists may face less mental fatigue because they practice at an academic center with lower volume compared to a community center with higher volume. Respondents also suggested the short trial duration, which limited colonoscopists’ familiarity with the module and ability to maximize its utility, could explain the results. Some respondents suggested they might have benefitted from additional introduction regarding the CADe module (eg, purpose, operating recommendations) to facilitate seamless integration into their practice and to minimize the impact on procedural time. Two alternative explanations, which are not supported by the data previously presented to colonoscopists,^20^ were that the positive impact of the division’s quality improvement efforts may have limited CADe’s ability to improve quality further and that aggregate results may obscure CADe’s potential benefit for lower-detecting colonoscopists.

## Discussion

The results of our pragmatic implementation trial of CADe with 24 unselected colonoscopists in routine clinical practice^20^ contrasted sharply with those of RCTs^21^, ^22^, ^23^ of the same U.S. Food and Drug Administration–approved CADe technology. These and other emerging real-world data^10^^,^^13^, ^14^, ^15^^,^^18^^,^^19^ that are discordant with the results of RCTs^7^, ^8^, ^9^ point to a gap between the effectiveness and efficacy of CADe. We are obliged to try to understand the underlying reasons for the contrasting results. In the current study, we achieved very high participation rates in surveys and interviews with the colonoscopists who participated in our pragmatic trial, aiming to gain insight into human-AI interactions.

The participants’ answers reflect high enthusiasm for CADe and trust for technology, including AI and CADe, which is consistent with the 97% CADe use rate that we observed in our pragmatic trial.^20^ However, the participants’ overall impression that CADe improved performance contrasts with the actual lesion detection results in our pragmatic trial, and was not consistent across all answers (only 10% believed that CADe found [or missed] a significantly meaningful number of polyps that they missed [or found], suggesting an impression that the ultimate level of detection was not affected much by CADe). When confronted with the pragmatic trial’s null results, most users expressed surprise about the collective lack of improvement but offered limited insight to explain the results. The participants’ erroneous and inconsistent impression of improved performance highlights the importance of measuring actual performance instead of relying on impressions of effectiveness, which may be unreliable.

In aggregate, the survey and interview answers make clear that our null pragmatic trial results cannot be explained by a lack of enthusiasm or lack of trust in CADe—in fact, the colonoscopists’ positive inclination might have been expected to increase the likelihood of positive results. However, the survey and interview answers do not provide a unifying compelling explanation for the pragmatic trial results. Still, respondents identified several factors that may be relevant to future technology development and dissemination. Although some colonoscopists characterized the “green box” and accompanying sound as distracting, colonoscopists expressed greater concern with the perceived frequency of false positives. Anecdotally, the number of false positives^32^ seemed manageable to us and unlikely to overwhelm or discourage colonoscopists. However, it is possible that colonoscopists may have dismissed true positive CADe prompts without careful appraisal, not distinguishing these from false positives.^20^ Additionally, the choice by several respondents to mute the CADe sound could have reduced the potential of CADe to affect detection rates, although it is unlikely that the remaining visual cue (“green box”) would have been missed.^33^ With polyps correctly detected by CADe, colonoscopists may have made errors in diagnosis and decisions about resection (ie, recognizing lesions detected by CADe but incorrectly diagnosing them as nonneoplastic and, thus, deciding not to resect). Despite the comments by some colonoscopists, it is not accurate that there was no room for improvement; although group detection rates in the pragmatic trial were high,^20^ one might have expected those in the lower baseline ADR tertiles to improve.

One potentially compelling issue raised by participants was the inability of CADe to correct procedural errors. In our survey, colonoscopists in the higher baseline ADR tertile were more likely than those in the other tertiles to believe that CADe may have led colonoscopists to relax on mucosal exposure. It is possible that, as a group, colonoscopists in our pragmatic study behaved differently from those in the RCTs across the multiple tasks that culminate in lesion removal, which is the basis for calculating all “detection” metrics. These include lesion detection, lesion diagnosis, deciding whether or not to resect, and performing resections—all of which depend on the foundation of good mucosal exposure. CADe aids directly in only the first task. This raises the question of whether CADe is best suited as a tool for improvement or as a means for increased efficiency and confidence in polyp identification.

Given that the CADe technology assessed in our pragmatic study and in randomized trials^21^, ^22^, ^23^ clearly identifies polyps in the colonoscopic field of view,^34^^,^^35^ it remains to be clarified which aspects of human-AI interaction explain the contrast between our results and those of RCTs with selected endoscopists. Our surveys and interviews offer some aggregate insight into this question (eg, enthusiasm and desire for the integration of AI technology, ease of use), but the individual insights into successful integration into clinical care are limited.

By design, we did not survey participants before they tried CADe because we wanted to avoid potentially influencing performance with priming questions. We acknowledge that our survey questions addressing beliefs and attitudes before trying CADe could, thus, be subject to recall bias. This was unavoidable. Similarly, the time between the end of the pragmatic trial and the survey and interview administration may have affected respondents’ memories of their experience with CADe and their ability to generate potential explanations for the trial results.

The choice to conduct our pragmatic trial rather than an RCT was deliberate. With several RCTs^21^, ^22^, ^23^ of the same U.S. Food and Drug Administration–approved CADe technology already completed, we lacked information about the effectiveness and generalizability of this technology in real-world, routine practice conditions. Pragmatic trials address a limitation of RCTs^36^^,^^37^—their limited ability to provide guidance on how to bring new technologies to patients. The embedded nature of pragmatic trials in a health care setting facilitates a better understanding of how and when a new technology works in real-world settings^38^^,^^39^ and reveals challenges to using the technology in practice. Even null results in the case of a pragmatic trial can provide valuable insights.^37^ Gaining insights about why pragmatic trial results may differ from RCTs, whether because of Hawthorne effects, lack of blinding, or other reasons, is critical to the design of more effective approaches to implementation in clinical practice.

The evolving literature on CADe has not yet identified a simple explanation for the contrasting results of RCTs and pragmatic trials. Mucosal exposure remains the critical foundation for polyp detection. CADe can only improve detection in exposed mucosa. We hypothesize that missing a polyp displayed on the screen may be a smaller problem than failing to expose mucosa and that in the positive RCTs, multiple dimensions may have been affected, including mucosal exposure—that is, that the improvement seen with CADe cannot be attributed exclusively to the computer detecting a polyp that the human eye did not see.

One potential limitation of our surveys is the number of respondents. However, we believe our response rate is much more important than the absolute number (21 and 22 out of the 24 colonoscopists in our CADe trial). Although a larger survey study of colonoscopists with a variety of exposures to CADe might provide different insights, the value of our mixed-methodology study derives precisely from its focus on a selected group of colonoscopists who had just completed a negative real-world pragmatic trial of CADe, the separation between Surveys 1 and 2 with intervening disclosure of the trial’s unexpected and disappointing results, and the very high response rates. We acknowledge that the ultimate number of participants was small and that the attitudes toward AI and CADe by our endoscopists in an academic division may not reflect those in other settings, such as community practice, or in other types of medical institutions. Whether our findings are generalizable can be tested in future studies with other colonoscopists.

The field of AI in endoscopy must now move forward in the context of the mixed results in the literature. In our pragmatic trial, we were interested in the real-world, open-label implementation impact of CADe, and we therefore made CADe available with a minimalist deployment strategy. However, how CADe is deployed might influence its results. Substantial research from organizational and implementation sciences^40^, ^41^, ^42^, ^43^ suggests that how units and unit managers deploy new technologies influence their uptake and outcomes. Attention to an implementation process, including intentionally planning for deployment, engaging clinicians in discussion about achieving the technology’s potential, and evaluating and reflecting after CADe’s initial deployment could also improve its use. It is possible that pragmatic CADe deployment with additional measures could reproduce the magnitude of improvement observed in RCTs. For instance, the inclination of endoscopists and human-AI interactions might differ from those in our pragmatic study if a clinical group has committed to the technology financially and, thus, has a vested interest in seeing it succeed; if deployment were accompanied by more intensive training in the application of the technology and its rationale; or if practice leaders explicitly set expectations that deployment of the technology should improve performance, particularly in those with lower performance at baseline.

Beyond CADe, it may take the development of a full suite of AI features^44^ to achieve the full potential of emerging technologies supporting endoscopy. These can include real-time assessment of mucosal exposure and prompts to ensure adequate inspection,^45^ sizing of lesions,^46^ computer-aided diagnosis,^47^, ^48^, ^49^ computer-aided assessment of resection adequacy,^50^ and support in generating endoscopy reports, which could in aggregate realize the promise of technology to optimize detection and resection rates by all endoscopists. Furthermore, failure to address underlying performance discrepancies between RCTs and pragmatic trials could undermine the progress of AI technology and the appropriate deployment of health care resources.

In conclusion, our surveys and interviews confirmed a positive inclination toward AI in endoscopy and a desire to include CADe in clinical practice among the colonoscopists who participated in our pragmatic trial of CADe, thus ruling out a lack of enthusiasm or mistrust of CADe as an explanation for the trial’s null results. The contrast between CADe’s efficacy in RCTs and its effectiveness in practice suggests that subtle aspects of the colonoscopist-technology interaction must be relevant, beyond the mere recognition by a computer of a “probable polyp” on the monitor screen that may have been missed by the human operator.

## Disclosure

The following author disclosed financial relationships: U. Ladabaum: Advisory board for UniversalDx, Lean Medical, Vivante, and Kohler Ventures and consultant for Medtronic, Clinical Genomics, Guardant Health, Freenome, and ChecCap. All other authors disclosed no financial relationships. This work was supported by a grant from the National Science Foundation (2026498).

## Figures and Tables

**Supplementary Table 1:** What colonoscopists liked and disliked about the CADe module

Responses	Example response
Colonoscopists liked	
CADe’s ability to detect flat polyps	“I liked its detection of flat polyps on the left colon, It found lesions I may not have seen.” (ID 10)
CADe’s ability to track polyps	“When a small polyp was found and you lost your position, GI Genius is very effective at locating the small polyp again, particularly for flat polyps, sessile serrated polyps.” (ID 29)
Feeling encouraged to conduct a thorough examination	“Made me more aware of the importance of a thorough examination/visualization of the mucosa.” (ID 24)
Feeling comfort/support from “second pair of eyes”	“In fact I felt it offered an extra layer of comfort to know that we are being thorough (like having a second opinion or an extra set of eyes, which helps when one is always worried about missing a polyp, like me).” (ID 21)
Ease of use	“easy to use, fast—real-time” (ID 11)
Colonoscopists disliked . . .	
Frequency of false positives of a polyp	“I disliked its overcalling of polyps due to poor prep or bubbles.” (ID 10)
CADe’s impact on procedure length	“Makes the procedure long.” (ID 22)
Variability in detection based on polyp type	“It has trouble with larger or more subtle polyps. It might miss them if the endoscopist is not already aware of them; ie, sometimes the green box appeared only after I focused on an area at the right distance, etc.” (ID 28)

Source: analysis of Survey 1 responses to open-ended questions: “What did you LIKE about the CADe (GI Genius) module?” and “What did you DISLIKE about the CADe (GI Genius) module?”

*CADe*, Computer-aided detection; *ID*, identifier.

**Supplementary Table 2:** Potential uses of AI in clinical practice and in endoscopy

Potential use	Quote
Generating clinic visit note and precharting	“Scribing a clinic note, precharting and gathering critical information before a clinic visit.” (ID 26)
Detecting pathophysiology outside of polyps	“The use of AI for detections of pathophysiology outside of polyps would be nice. For instance, IBD staging or detection of microscopic colitis.” (ID 10)
Responding to voice commanded functions	“Order functions to aid in endoscopy, like Siri: ‘take picture,’ ‘NBI,’ ‘near-focus,’ ‘start video recording’ (would help in case of a difficult maneuver or when hands are busy).” (ID 21)
Characterizing preparation/procedure	“Characterize polyp (hyperplastic, serrated, adenoma) by pit pattern.” (ID 29)

Source: analysis of Survey 1 questions: “What other kinds of assistance, if any, would Artificial Intelligence (AI) be useful for in clinical practice in general?” and “Do you have suggestions about other possible uses of Artificial Intelligence (AI) specifically in endoscopy?”

*AI*, Artificial intelligence; *IBD*, inflammatory bowel disease; *ID*, identifier; *NBI*, narrow-band imaging.

**Supplementary Table 3:** Rationale for CADe trial results at Stanford

Response category	Quote
Patient cohort	“The patient cohort at Stanford: potentially younger than average, potentially better educated and thus on top of health surveillance, potentially better prepped?” (ID 11)
High ADR versus national standards	“Using tools like GI Genius do not have added benefit at centers that already have high polyp detection rates.” (ID 12)
Removal of sound	“Green box became part of the background. I found sound disruptive and turned it off. If sound kept on would performance be better?” (ID 20)
Inability to rectify procedural errors	“I think the most important factor is mucosal exposure—instead of missing polyps on the monitor (which is where GI Genius could help). If mucosal exposure did not change (either improving or worsening) during the GI Genius trial, then it would make sense that detection rates did not change much.” (ID 28)

Source: analysis of Survey 2 question: “What do you think best explains the results of the trial of the CADe (GI Genius) module at Stanford?”

*ADR*, Adenoma detection rate; *CADe*, computer-aided detection; *ID*, identifier.

**Supplementary Table 4:** Experiences with the CADe module

Nature of experience	Description	Quote
Factors detracting from CADe’s value	Frequency of false positives	“The negative part of it to me was the number of, in my opinion, false positive alerts, which at one point got a little tiring to recheck. . . . I think that the system needs some more refinement. . . . I mean, for one colonoscopy, it’s fine. If it’s like getting more, then it’s almost overwhelming, because you are getting too many. . . . And at one point you [are] completely overwhelmed. Basically your attention span is just exhausted. So at one point . . . I told them, okay, just turn it off for the next two or so, just get a break because . . . of course, we are like worried and then go back and look. . . . Then it delays the procedure, and it is . . . tiring. . . . I want to give my brain a break.” (ID 11)
	Prolonged procedure time during acclimatization	“I had a hard time initially getting used to using it. There were a lot of false polyps that I think it picks up. So that took a little while getting used to it. It prolonged my procedure time I will say, definitely, which was sort of unexpected. So, there is a learning curve to it.” (ID 19)
	Distractors	“The most annoying thing in the world was the little alarm that was beeping. . . . Every time you saw a bubble . . . the box would appear, and there would be an alarm. And that caused alarm fatigue very quickly, and it was very frustrating. After, like 1 or 2 procedures, I had to turn that alarm off. But I don’t find that the box, the green box that appeared on a nonpolyp to be annoying if there was no sound because I’m looking anyways, so that didn’t seem to bother me as much.” (ID 23)
Neutral factors of CADe	Neither improved nor inhibited experience: Who saw the polyp first?	“It’s hard to know [if it helped me perform better] because did my eyes automatically go to the lesion of interest or did my eyes go to that area because the green box was around it, and it’s happening in fractions of a second. It’s hard to determine. That’s why I think it’s hard as an individual to know if it is helpful because did I see it or did I only see it because the box was there? . . . Were there lesions that there’s absolutely no chance I would’ve seen without the box? That would be harder for me to be confident about. There may have been one or two, but that was not the dominant part of my experience.” (ID 16)
Factors enhancing the value of CADe	Ability to stabilize a scope	“But you find a little polyp in an area where the colon is maybe contracting actively the scope isn’t stable . . . maybe you lose it. There’s nothing more frustrating than that when you’re dealing with a small polyp, because in your heart of hearts, you know it’s probably not that clinically significant. On the other hand, to have seen it, and not taken it out, that’s just totally substandard. So you end up hunting for it, and it’s a waste of time, effort. It’s aggravation, and in those situations sometimes when I would, you know, be passing the snare, or inadvertently the scope moves, or something happens, and you kind of lose it. The [GI-Genius] would continue tracking it, and even if your orientation flipped, as long as it was in the field of view, it was there, so in that way it’s definitely helpful. Once it’s found something, it tends not to lose it, and if you do come out of frame and then back in, it’ll find it readily.” (ID 28)
	“Comfort” provided by the module provided confidence in physician’s skill	“I enjoyed the AI program because it was kind of like a challenge for me, to up my ante and be as good as the machine. So making, you know, every time the green box lit up, I was comparing whether I had already noticed it. So that was a fun personal challenge of seeing, I guess how good my skills are, and over the weeks, or couple of months that we did this, I got better at it. The other nice part I liked about it was having sort of a second set of eyes helping me with the exam, and making sure I didn’t miss anything.” (ID 24)

Source: analysis of interviews about CADe technology and results.

*AI*, Artificial intelligence; *CADe*, computer-aided detection; *ID*, identifier.

**Supplementary Table 5:** Endoscopists’ rationale for CADe trial results

Rationale category	Respondents by ADR tertile	Quote
High physician skill/baseline ADRs at Stanford resulting in little room to improve by CADe	n = 4 bottom n = 3 middle n = 2 top	“I think there’s a ceiling effect within our group. . . . I think we’re pretty good at finding polyps, and so technology that will help find more polyps, you can only find so many polyps. I think that’s one problem.” (ID 23)
Short duration of trial prevented familiarity with the module	n = 2 bottom n = 1 top	“No, I remember being worried that maybe it would make me pick up tiny, tiny things that were not significant, and that I would spend a lot more time, because basically, whenever it’s picked up something, you have to go and look at it and wash it. But I don’t, you know. I guess we didn’t have enough experience with it that it really became a reality. But I feel like if we had used it for a much longer period of time that might be something that would be kind of.” (ID 20)
CADe is unable to correct procedural errors (eg, insufficient opening of mucosa)	n = 1 middle n = 2 top	“So you know, in order to find a polyp, there’s a couple of different things that have to happen. First, you have to get to the end of the colon. Technology isn’t gonna help with that. Then you have to clear the mucosa, meaning you have to suction all the fluid out. You have to appropriately wash the colon, the technology isn’t going to help with that. You also have to open the colon, so that you’ve adequately the spread things with technical skill. The technology isn’t going to help with that. So, the only thing that technology really helps with is identifying, is correct recognition of pathology, meaning that you can see a lesion and recognize that it’s a polyp. And that is very practice dependent; that is skill dependent. And so for some providers it’s not going to probably make a huge difference. For other providers, it will make a difference.” (ID 23)
Educated and health-conscious patient population	n = 2 bottom	“So I thought first of all, so I mean the patient cohort at Stanford may or may not be like a special one, so we have a lot of patients who . . . are highly educated. They’re potentially taking care of their health. I mean, they are very often like engineers at Google . . . Facebook, Apple. They’re well-educated people who potentially take care more of their health and really follow up on preventative things. They have good health insurance, et cetera. So that means as well. The people who have like follow-up colonoscopies. Um, you know they are better prepped. They are potentially having less polyps compared to other areas, because they are like, very like good with their stuff. Maybe the age of the patients. I don’t know if that’s the case. The preparation is a key thing, and like the quality of the detection, so like, how better the pro like the preparation is the better the um detection is obviously, and if you have a person who’s like having a college or higher degree, doctoral degree, one would normally assume they’re like, following these instructions better, and they are maybe having a healthier lifestyle as well, having maybe less polyps or so on all looking, you know. Then, following up the five years procedure, so it helps definitely to like having a better quality procedure itself.” (ID 11)
Existing QI efforts at Stanford have led to higher detection rates and less room for improvement	n = 1 bottom n = 1 top	“I also think that with the program that [head of division] has instituted, I am more systematic. Like I have developed ways that I perform things to make sure that, kind of inside checks, make sure that I do certain things to satisfy myself that I did the best job that I could. And getting that grade every quarter, say like this is how many polyps you detected. You take it to heart. Yeah. So I think that we’re constantly improving, and probably that was the biggest factor I would think.” (ID 20)
Potential misinterpretation of green box as a “false positive” resulting in incorrect dismissal	n = 1 top	“Yes, GI Genius pointed some out, but people were more careless in polyps that GI Genius didn’t box, but I don’t think GI Genius is missing many polyps, so I don’t think that’s the likely explanation. Could it be that the GI Genius is boxing things that they then look at and say, “Wait a minute. That’s not really a polyp,” and they’re just wrong, where GI Genius is right and they’re wrong in assessing it as a false positive. They don’t resect it, and therefore it can’t count as a detected adenoma, and it’s a missed adenoma. That could be also, and I don’t know what is attributable to which of these factors. But it’s exposure. It’s a misinterpretation of the green box potentially.” (ID 28)

Source: analysis of interviews about CADe technology and results.

*ADR*, Adenoma detection rate; *CADe*, computer-aided detection; *QI*, quality improvement.

**Table:** 

1	2	3	4	5	6
Most					Least
Valuable					valuable

**Table:** 

1	2	3	4	5	6
Most					Least
Valuable					valuable

**Table:** 

1	2	3	4	5	6
Most					Least
Valuable					valuable

**Table:** 

1	2	3	4	5	6
Most					Least
Valuable					valuable

**Table:** 

1	2	3	4	5	6
Most					Least
Valuable					valuable

**Table:** 

1	2	3	4	5	6
Most					Least
Valuable					valuable

## Full methodology

### Study design and setting

We used a sequential, mixed-methodology design^23^ to pursue an understanding of Stanford colonoscopists’ experience with CADe (GI Genius Intelligent Endoscopy Module, Medtronic, Minneapolis, Minn, USA) and to explore potential explanations for the lack of improvement in quality metrics with the use of CADe in our pragmatic trial.^18^ We had not surveyed colonoscopists before or during the pragmatic trial given our explicit intent to avoid any undue influence on performance. Stanford University’s institutional review board approved this study under expedited procedures.

We developed and administered 2 surveys, the first after the pragmatic trial (Survey 1) and the second after the disclosure of our pragmatic trial results at a faculty meeting (Survey 2). We then developed an interview guide and conducted qualitative interviews that built on the survey results to gain a deeper understanding of colonoscopists’ reactions and to explore potential explanations for the pragmatic trial results.

### Study participants

We invited the 24 colonoscopists who participated in the CADe pragmatic trial^18^ from February 16 through May 13, 2022, to complete the 2 surveys and the interviews. Participation was voluntary, and respondents received no remuneration. Our sample included all the colonoscopists who participated in our pragmatic trial. We included everyone exposed to CADe as part of the pragmatic trial to be as rigorous as possible because this was one of the first studies to show disappointing real-world results compared to RCTs.

### Surveys 1 and 2

We developed our surveys based on the “Survey on the Future of Technology-Assisted Work,” which social scientists developed previously to study beliefs and attitudes toward technology in the intensive care setting (survey available upon request).

Survey 1 included 33 questions probing attitudes and beliefs about CADe. For most questions, respondents used a 5-point Likert scale to indicate the extent of their agreement with statements concerning the utility of AI in endoscopy and trust in AI before and after trying CADe as well as their experience using CADe. The survey also included 1 true/false question, 6 questions asking respondents to rank order 6 potential roles for AI in endoscopy from 1 (most valuable) to 6 (least valuable), and 4 open-ended questions regarding likes and dislikes about the CADe module and other kinds of assistance for which AI could be useful in general practice and in endoscopy specifically.

Survey 2 included 8 questions about colonoscopists’ reactions to and explanations for the CADe pragmatic trial results. One assessed the degree of surprise, if any, over the results, and 5 explored the extent to which respondents agreed with potential explanations for the results, using a 5-point Likert scale. The last 2 items were open-ended inquiries of potential alternative explanations for the contrasting pragmatic trial versus RCT results.

### Survey administration

One author (U.L., who serves as clinical chief of the Division of Gastroenterology and Hepatology) invited colonoscopists to participate via individual e-mails, including multiple individual and group reminders.

We administered Survey 1 from September 15 through October 11, 2022. On October 12, 2022, the pragmatic trial results were disclosed at a faculty meeting, without group discussion so as not to influence the results of Survey 2 or subsequent interviews. Colonoscopists requested no clarification, suggesting they understood the null results. We administered Survey 2 from October 18 through November 13, 2022. The social scientist collaborators conducted individual interviews via Zoom (Zoom Video Communications, San Jose, Calif, USA) from November 15, 2022, through January 20, 2023.

Based on our quality assurance program’s routine comprehensive audit,^24^, ^25^, ^26^, ^27^ we divided the 24 colonoscopists into tertiles based on their baseline ADR performance in the 12 months preceding the CADe pragmatic trial.^18^ Colonoscopists were assigned blinded identifiers, and the key was not disclosed to other authors and was not consulted again, as described previously.^25^

### Survey data analysis

For most items, responses were recorded on a 5-point Likert scale for the degree of respondent agreement to each item, ranging from strongly disagree (1) to strongly agree (5). For each item, we calculated the percentage of responses for each Likert scale option. Descriptive results were reported using stacked bar graphs, focusing on the percentage of responses indicating agreement and strong agreement. For items in Survey 1 that were asked twice, before and after trying CADe, the differences in responses were calculated and compared using the Wilcoxon signed rank test. For items ranking potential roles for AI systems in endoscopy, the mean and standard deviation were reported. We linked deidentified, coded survey data with the corresponding baseline ADR tertile for each respondent and compared results by tertile using the Kruskal-Wallis test. Complete case analysis was performed. A *P* value of <.05 was considered statistically significant as a descriptive analysis. The analysis was performed using SAS software version 9.4 (SAS Institute Inc, Cary, NC, USA).

### Interview guide, data collection, and analysis

Building on survey findings, we developed a semistructured interview guide, consisting of 8 questions, to probe endoscopists’ reactions to the use of CADe in endoscopy and to explore potential explanations for the trial results. The interviewers were trained members of the social science research group, not affiliated with the clinical unit, and they remained blinded to respondents’ Survey 1 and 2 responses and performance during the CADe trial. With participant consent, they recorded and transcribed the interviews.

The social science team deidentified the transcripts and analyzed the data according to principles of thematic analysis, combining deductive and inductive approaches.^28^ First, the researchers completing the coding familiarized themselves with the data by reviewing all transcripts. After the initial review, the social science team met to discuss and generate an initial set of codes, which were deduced based on the research questions, interview guide, and their understanding of the data. These fit 3 overarching categories: colonoscopists’ experience with CADe, colonoscopists’ explanations for trial results, and colonoscopists’ feedback on the pragmatic trial. Next, 2 coders used NVivo qualitative software (Lumivero, Denver, Colo, USA) to code the interviews. They searched for excerpts associated with the coding categories and inductively identified subcodes emerging from the data during the coding process. During this coding process, the social science team met to review and revise the application of subcodes and to resolve any discrepancies in the interpretation and analysis of the data.

After completing the coding process, deidentified interview participant codes were linked with participants’ coded baseline ADR tertile, allowing the social science team to compare interview results by baseline ADR tertile.

Survey 1: Respondent attitudes before disclosure of the trial results

Thank you for participating in this brief survey regarding Artificial Intelligence (AI) and computer-aided detection (CADe) of polyps at colonoscopy.

This survey is open to all endoscopists who participated in the open-label trial of Medtronic GI Genius technology in the Redwood City Outpatient Procedure Center from February 2022 through May 2022.

For each item, please reflect on your beliefs and attitudes **BEFORE trying the computer-aided detection (CADe) module (GI Genius) for polyp detection**, and select the best answer for you.

To what extent do you agree or disagree with the following?

1. BEFORE trying the CADe (GI Genius) module, my experience with new technologies at work generally led me to expect that technology will support rather than hinder my work.
  1. Strongly agree
  2. Agree
  3. Neither agree nor disagree
  4. Disagree
  5. Strongly disagree
2. BEFORE trying the CADe (GI Genius) module, I was enthusiastic about the application of artificial intelligence (AI) in endoscopy.
  1. Strongly agree
  2. Agree
  3. Neither agree nor disagree
  4. Disagree
  5. Strongly disagree
3. BEFORE trying the CADe (GI Genius) module, I believed that applying artificial intelligence (AI) in endoscopy is in the best interest of patients.
  1. Strongly agree
  2. Agree
  3. Neither agree nor disagree
  4. Disagree
  5. Strongly disagree
4. BEFORE trying the CADe (GI Genius) module, I trusted the CADe (GI Genius) module to detect polyps that are displayed on the screen.
  1. Strongly agree
  2. Agree
  3. Neither agree nor disagree
  4. Disagree
  5. Strongly disagree
5. BEFORE trying the CADe (GI Genius) module, I regarded my abilities in endoscopy as a critical part of my professional identity.
  1. Strongly agree
  2. Agree
  3. Neither agree nor disagree
  4. Disagree
  5. Strongly disagree

For each item, please reflect on your beliefs and attitudes **AFTER trying the computer-aided detection (CADe) module (GI Genius) for polyp detection**, and select the best answer for you.

To what extent do you agree or disagree with the following?

6. AFTER trying the CADe (GI Genius) module, I believe this technology can support rather than hinder my work.
  1. Strongly agree
  2. Agree
  3. Neither agree nor disagree
  4. Disagree
  5. Strongly disagree
7. AFTER trying the CADe (GI Genius) module, I am enthusiastic about the application of artificial intelligence (AI) in endoscopy.
  1. Strongly agree
  2. Agree
  3. Neither agree nor disagree
  4. Disagree
  5. Strongly disagree
8. AFTER trying the CADe (GI Genius) module, I believe that applying artificial intelligence (AI) in endoscopy is in the best interest of patients.
  1. Strongly agree
  2. Agree
  3. Neither agree nor disagree
  4. Disagree
  5. Strongly disagree
9. AFTER trying the CADe (GI Genius) module, I trust the CADe (GI Genius) module to detect polyps that are displayed on the screen.
  1. Strongly agree
  2. Agree
  3. Neither agree nor disagree
  4. Disagree
  5. Strongly disagree
10. I received adequate training on the use of the CADe (GI Genius) module.
  1. Strongly agree
  2. Agree
  3. Neither agree nor disagree
  4. Disagree
  5. Strongly disagree
11. The CADe (GI Genius) module was easy to use.
  1. Strongly agree
  2. Agree
  3. Neither agree nor disagree
  4. Disagree
  5. Strongly disagree
12. The CADe (GI Genius) “green boxes” when there was not really a polyp were bothersome.
  1. Strongly agree
  2. Agree
  3. Neither agree nor disagree
  4. Disagree
  5. Strongly disagree
13. The CADe (GI Genius) sound that went along with the “green box” was bothersome.
  1. Strongly agree
  2. Agree
  3. Neither agree nor disagree
  4. Disagree
  5. Strongly disagree
14. I eventually turned off the CADe (GI Genius) sound that went along with the “green box.”
  1. True
  2. False
15. The CADe (GI Genius) module improved my overall performance as a colonoscopist.
  1. Strongly agree
  2. Agree
  3. Neither agree nor disagree
  4. Disagree
  5. Strongly disagree
16. The CADe (GI Genius) module FOUND a clinically meaningful number of polyps that I missed.
  1. Strongly agree
  2. Agree
  3. Neither agree nor disagree
  4. Disagree
  5. Strongly disagree
17. The CADe (GI Genius) module MISSED a clinically meaningful number of polyps that I found.
  1. Strongly agree
  2. Agree
  3. Neither agree nor disagree
  4. Disagree
  5. Strongly disagree
18. The CADe (GI Genius) module improved my lesion detection rates during colonoscopy.
  1. Strongly agree
  2. Agree
  3. Neither agree nor disagree
  4. Disagree
  5. Strongly disagree
19. The CADe (GI Genius) module made me focus on exposing all the colonic mucosa better.
  1. Strongly agree
  2. Agree
  3. Neither agree nor disagree
  4. Disagree
  5. Strongly disagree
20. What did you **LIKE** about the CADe (GI Genius) module?
21. What did you **DISLIKE** about the CADe (GI Genius) module?

For each item, please reflect on your beliefs, attitudes and wishes **FOR THE FUTURE**, and select the best answer for you.

To what extent do you agree or disagree with the following?

22. I would like to have the CADe (GI Genius) module that we trialed available for all my colonoscopies.
  1. Strongly agree
  2. Agree
  3. Neither agree nor disagree
  4. Disagree
  5. Strongly disagree
23. I would like to have Artificial Intelligence (AI) applications available for all my colonoscopies.
  1. Strongly agree
  2. Agree
  3. Neither agree nor disagree
  4. Disagree
  5. Strongly disagree
24. I am concerned that monitoring colonoscopy quality metrics may be used against me.
  1. Strongly agree
  2. Agree
  3. Neither agree nor disagree
  4. Disagree
  5. Strongly disagree
25. I worry that technology will replace me in doing important aspects of my work.
  1. Strongly agree
  2. Agree
  3. Neither agree nor disagree
  4. Disagree
  5. Strongly disagree

Listed below are **6 potential roles** for Artificial Intelligence (AI) systems in endoscopy. Please **RANK** these potential roles from 1 (most valuable) to 6 (least valuable) based on the value that you would attach to each, assuming its benefit has been established through credible scientific research. Please use each ranking (i.e., 1, 2, 3, 4, 5 and 6) ONLY ONCE.

26. Assisting clinicians by predicting a patient’s clinical course or outcome (e.g., cancer risk stratification, or predicting risk of endoscopic adverse events).

27. Assisting clinicians with ensuring adequate mucosal exposure during colonoscopy, through real-time feedback.

28. Assisting clinicians with ensuring adequate polyp detection during colonoscopy, through real-time feedback.

29. Assisting clinicians with ensuring adequate polyp characterization (e.g., adenoma) during colonoscopy, through real-time feedback to guide real-time decisions about polyp resection.

30. Assisting clinicians with ensuring adequate polyp size determination during colonoscopy, through real-time feedback.

31. Assisting clinicians by automatic documentation of clinical activities (e.g., endoscopy report generation).

32. What other kinds of assistance, if any, would Artificial Intelligence (AI) be useful for in clinical practice in general?
33. Do you have suggestions about other possible uses of Artificial Intelligence (AI) specifically in endoscopy?

Survey 2. Respondent attitudes after disclosure of trial results.

For each item, please reflect on your beliefs and attitudes **AFTER LEARNING THE RESULTS** of the Stanford trial of the computer-aided detection (CADe) module (GI Genius) for polyp detection, and select the best answer for you. As a reminder, three randomized controlled trials of GI Genius have shown higher adenoma detection rates and lower adenoma miss rates in the GI Genius arm vs. the control (non-GI Genius) arm. In contrast, the detection rates during our open-label GI Genius trial at Stanford were comparable to those without use of GI Genius (historical control, and concurrent control at sites other than Redwood City Outpatient Procedure Center).

To what extent do you agree or disagree with the following?

1. I am surprised by the results of the trial of the CADe (GI Genius) module at Stanford.
  1. Strongly agree
  2. Agree
  3. Neither agree nor disagree
  4. Disagree
  5. Strongly disagree
2. I believe the results of the trial of the CADe (GI Genius) module at Stanford represent a chance outlier (e.g., maybe we colonoscoped people with fewer-than-average polyp numbers during the trial period).
  1. Strongly agree
  2. Agree
  3. Neither agree nor disagree
  4. Disagree
  5. Strongly disagree
3. A possible explanation for the results of the trial of the CADe (GI Genius) module at Stanford is that the CADe module did not identify a substantial number of polyps that might have otherwise been missed.
  1. Strongly agree
  2. Agree
  3. Neither agree nor disagree
  4. Disagree
  5. Strongly disagree
4. A possible explanation for the results of the trial of the CADe (GI Genius) module at Stanford is that the CADe module led endoscopists to relax in their effort to expose all mucosa well, instead relying on the CADe module to detect polyps.
  1. Strongly agree
  2. Agree
  3. Neither agree nor disagree
  4. Disagree
  5. Strongly disagree
5. A possible explanation for the results of the trial of the CADe (GI Genius) module at Stanford is that the “green box” appeared so often that endoscopists may have ignored it.
  1. Strongly agree
  2. Agree
  3. Neither agree nor disagree
  4. Disagree
  5. Strongly disagree
6. A possible explanation for the results of the trial of the CADe (GI Genius) module at Stanford is that when the “green box” identified bumps as “possible polyps,” endoscopists almost always decided those were not polyps, and therefore did not remove them.
  1. Strongly agree
  2. Agree
  3. Neither agree nor disagree
  4. Disagree
  5. Strongly disagree
7. What do you think best explains the results of the trial of the CADe (GI Genius) module at Stanford?
8. How should we interpret the fact that the CADe (GI Genius) module did not improve lesion detection rates in our trial at Stanford, but it did in several randomized controlled trials?

Interview guide

1) What was your experience with the CADe (GI Genius) module? Probe for:
  a. Did you regard it as a positive / negative / neutral experience? Why?
  b. Did you perceive any benefits or challenges (e.g., logistical issues) with GI Genius that impacted your use of it?
2) How do you think CADe (GI Genius) module was supposed to help you perform colonoscopy better?
3) Were there instances where you felt that the CADe (GI Genius) module was, in fact, helping you perform better? Can you share an example?
4) Were there instances where you felt it was inhibiting your performance? An example here would be great too.
5) Why do you think the CADe (GI Genius) module did not improve lesion detection rates at a group level?
6) What could you imagine would make using the CADe (GI Genius) module improve lesion detection rates? Probe for: anything about the way it was implemented or the way you think it might be being used?
7) Any impressions or thoughts that you would like to share regarding the CADe (GI Genius) **module** itself?
8) Any impressions or thoughts that you would like to share regarding the **trial** or the implementation of the CADe (GI Genius) module at Stanford?
